# Detection of distinct MERS-Coronavirus strains in dromedary camels from Kenya, 2017

**DOI:** 10.1038/s41426-018-0193-z

**Published:** 2018-11-28

**Authors:** Stella Kiambi, Victor M. Corman, Rina Sitawa, Jane Githinji, James Ngoci, Abdullahi S. Ozomata, Emma Gardner, Sophie von Dobschuetz, Subhash Morzaria, Joshua Kimutai, Simon Schroeder, Obadiah Njagi, Piers Simpkin, Gabriel Rugalema, Zelalem Tadesse, Juan Lubroth, Yilma Makonnen, Christian Drosten, Marcel A. Müller, Folorunso O. Fasina

**Affiliations:** 1 Food and Agriculture Organization of the United Nations (FAO), Rome, Italy; 2 Charité-Universitätsmedizin Berlin, corporate member of Freie Universität Berlin, Humboldt-Universität zu Berlin, and Berlin Institute of Health, Institute of Virology, Berlin, Germany; 3 German Centre for Infection Research, associated partner Charité, Berlin, Germany; 4 Directorate of Veterinary Services, Nairobi, Kenya; 5 University of Nairobi, Nairobi, Kenya

Dear Editor,

MERS-Coronavirus (CoV) is a dromedary-transmitted zoonotic pathogen that is associated with severe viral pneumonia in humans^1^. As of 28 September 2018, 2249 infections and 798 fatalities (36%) from 27 countries had been reported to the World Health Organization^2^. Although the majority of dromedaries are found in Africa^3^, zoonotic spillover events, nosocomial outbreaks, and human fatalities occurred predominantly in the Arabian Peninsula^2^. Recently identified MERS-CoV strains from Egyptian and Ethiopian dromedaries differed genetically and phenotypically from MERS-CoV strains on the Arabian Peninsula^4,5^. In 2017 we identified and characterized two independently circulating MERS-CoV strains in two dromedary herds in Kenya.

Kenya is located within the Greater Horn of Africa, a region that hosts 80% of the world's dromedary camel population, exporting up to 300000 dromedaries to the Arabian Peninsula per year^3^. Our previous sero-epidemiological studies showed that MERS-CoV is widespread in Kenyan dromedaries^6^ and that autochthonous human MERS-CoV infections may occur^7^. To date we acknowledge on genotypic or phenotypic traits of MERS-CoV strains in Kenya.

Between July 2016 and October 2017, nasal swabs were randomly taken from *n* = 1421 dromedaries in five counties, namely, Turkana (*n* = 417), Marsabit (*n* = 370), Isiolo (*n* = 403), Laikipia (*n* = 181), and Nakuru (*n* = 50). In addition, monthly repeated sampling was performed on 430 dromedaries from four herds in two different countries (Isiolo and Nakuru) for a period of 7 months (from April to October 2017). In total, *n* = 2175 nasal swab samples were collected. All samples were stored frozen in TRIzol buffer at −80 °C. RNA extraction (Direct-zol™ RNA kit, Zymo Research) and MERS-CoV nucleic acid detection were performed following the manufacturer's instructions and according to previously established protocols^8^.

In seven of 2175 (0.23%) tested nasal swabs, MERS-CoV RNAs were detected by the  upE MERS-CoV RT-PCR screening assay (Supplementary Table). For 2/7 samples, which had very low MERS-CoV RNA concentrations (<2 x 10^4^ copies/ml), confirmatory RT-PCR testing and sequencing were unsuccessful. The mean viral load for 5/7 samples was 1.1 x 10^7^ (range 1.2 x 10^5^–5.0 x 10^7^) RNA copies per ml buffer. Four of the five MERS-CoV RNA-positive animals were female and <1 year old, consistent with previous observations that juvenile dromedaries and possibly females may be the main sources for MERS-CoV excretion^9^. The MERS-CoV RNA-positive animals belonged to two different dromedary camel herds in Dabel and Lombolio, which are both located within Isiolo country. However, the herds neighbor each other and share common pastures and water sources. During the time of the study, there were no new camels introduced into the two herds. The dromedaries were sampled on the same day, suggesting simultaneous cocirculation of two different MERS-CoV strains and perhaps an unexplored infection dynamic in Isiolo, which is a camel congregation location. To experimentally confirm the presence of two independently circulating MERS-CoV strains and to rule out sample cross-contamination, we generated complete MERS-CoV genome sequences using a previously established protocol^10^. Full genome sequences were generated for one specimen of each of the two positive herds using the samples with the highest MERS-CoV RNA concentrations (5.0 x 10^7^ and 3.7 x 10^6^ copies/ml). Other confirmed MERS-CoV-positive samples were assigned to two different MERS-CoV isolates (“Dabel” or “Lombolio”) by amplifying and sequencing single-nucleotide polymorphisms in the spike gene and the open reading frame 3 (Supplementary Table). All three viruses from Dabel and both viruses from Lombolio shared the same polymorphism patterns.

For phylogenetic analysis, we included two representatives of MERS-CoV lineages representing MERS-CoV clades A and B, as defined earlier^11^, along with all published clade C (non-A, non-B) MERS-CoV complete genomes (GenBank accessed 2 April 2018). A phylogenetic tree was constructed using the maximum-likelihood method based on the general time reversible model and 500 bootstrap replicates using the PhyML plugin in Geneious R11 (www.geneious.com, Biomatters Ltd, New Zealand).

As shown in Fig. 1, both Kenyan dromedary MERS-CoV isolates clustered with the proposed clade C viruses from Ethiopia and Egypt in a sister relationship to all Arabian MERS-CoVs (clades A and B). The two Kenyan MERS-CoV isolates diverged by 0.02% at the nucleotide level, confirming the circulation of at least two different MERS-CoV strains in Kenya. The next closest MERS-CoV relative was obtained from a dromedary sampled in Egypt in 2014 (NRCE-NC163/2014; Acc No. KU74020, clade C) and showed 0.23–0.24% nucleotide distance. Recombination analysis by RDP V4.95 indicated that none of the two Kenyan MERS-CoV strains had recombined with any of the known clade A, B, or C strains.

**Fig. 1:**
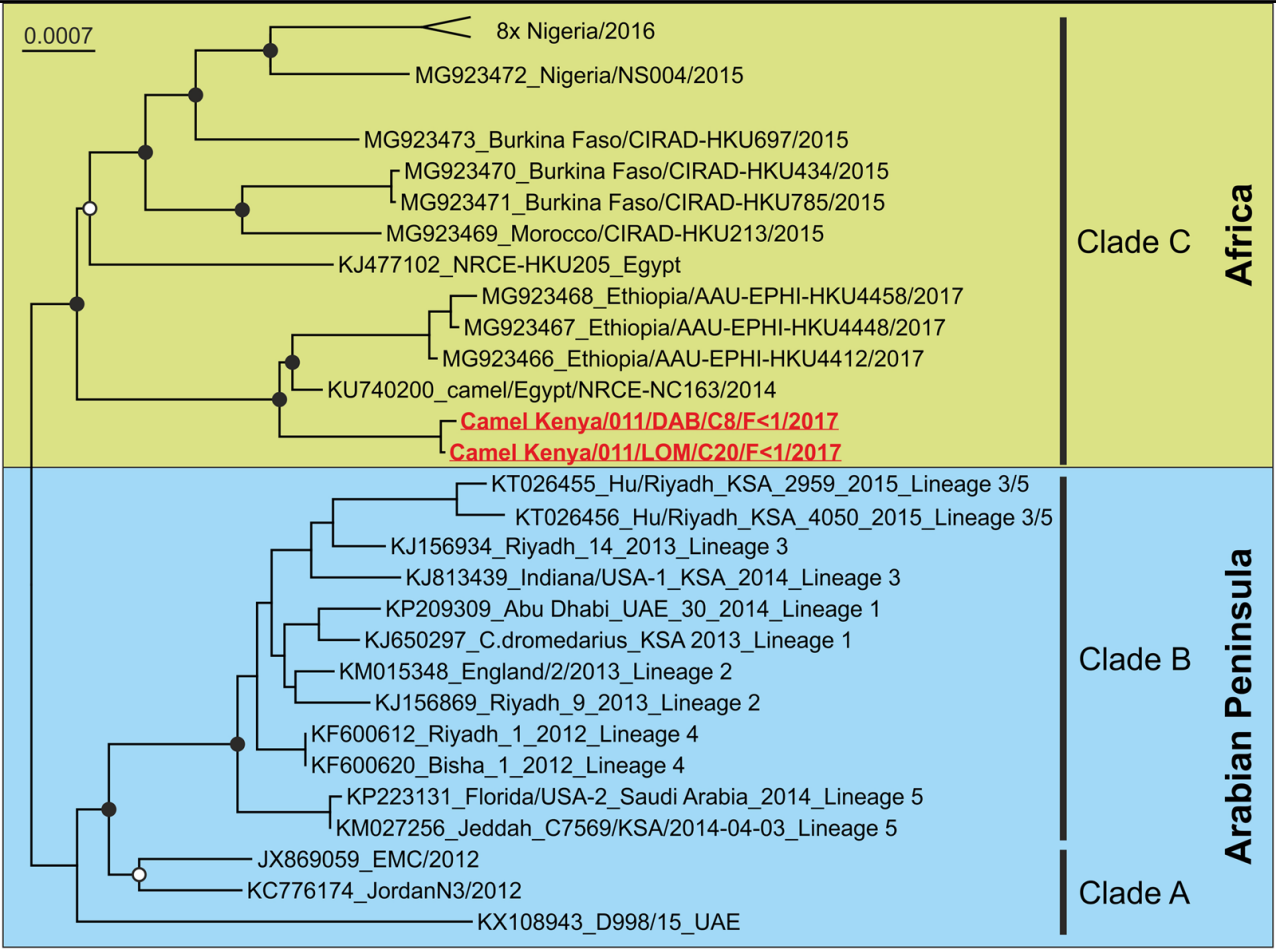
Maximum-likelihood phylogeny of the complete coding sequences of representatives of MERS-CoV and the two sequences from this study (highlighted in red). Virus designations include GenBank accession numbers and strain names. Black circles at nodes indicate bootstrap supports of >90% and white circles>75% (500 replicates). The top clade “8x Nigeria/2016” was collapsed for graphical reasons and contains eight MERS-CoV sequences (Acc. No. MG923474-81) from dromedaries in Nigeria in 2016

The previously described clade C African MERS-CoV strains^4,5^ had several mutations in the spike protein, which is responsible for cellular receptor interaction, virus entry, and antibody-directed virus neutralization^12^. An alignment of the amino-acid sequences of all known MERS-CoV spikes showed that the Kenyan MERS-CoV strains had one unique amino-acid change (S528P) within the core part of the receptor-binding domain (Supplementary Figure). As the mutation was not among the 14 amino-acid residues that directly interact with the dipeptidyl peptidase-4 receptor^12^, phenotypic traits of these new clade C MERS-CoV strains may be comparable to epidemic MERS-CoV strains as described previously^4^. However, without further extensive experimental assessment, we cannot rule out the possibility that the observed mutation in the spike protein causes differences in the receptor interaction or receptor binding affinity, which may influence virus transmission or host tropism.

Recently described MERS-CoV strains from Western Africa had genomic deletions within open reading frame (ORF) 4 a/b that were not seen in Eastern African MERS-CoV strains^4^. Both of the encoded proteins, proteins 4a and 4b, have anti-immune functions^13,14^ and may represent important virulence factors in vivo. We provide independent evidence that MERS-CoV from Eastern African dromedaries encode a complete ORF4a/b. The observation that MERS-CoV strains in different parts of Eastern Africa have a complete ORF4a/b suggests the predominance of these strains on the African continent and emphasizes that the ORF4a/b deletion is most likely geographically restricted to Western Africa.

Taken together, differential spike-receptor interactions and anti-immune activity may influence virus replication and transmission. The limited number of human MERS cases in Africa would certainly favor the idea that MERS-CoV strains differ in virulence and transmissibility. Further experimental confirmation, preferably by animal transmission experiments in combination with coronavirus reverse genetics^15^, are warranted.

The phylogenetic relationship of MERS-CoV strains from the African continent (clade C) with the strains circulating on the Arabian Peninsula (clades A and B) hints at the divergence of these clades some time ago. The putative absence of clades A and B MERS-CoVs on the African continent may be explained by a lack of surveillance and testing and/or by the genetic drift of MERS-CoV on the Arabian Peninsula. The unidirectional export routes from Africa to the Arabian Peninsula may prevent the reintroduction opportunities of clades A and B MERS-CoVs into African dromedary herds. Interestingly, to date, no clade C MERS-CoV strains from Africa have been detected on the Arabian Peninsula, which is rather surprising, given the continuous and extensive export of African dromedaries to the Arabian Peninsula. An explanation for this observation may again be a lack of testing of imported animals and/or the fact that previous clade A/B MERS-CoV infections may have established herd immunity in the Arabian dromedary populations. As CoV infections do not elicit long-lasting (mucosal) immunity, the introduction of clade CMERS-CoV strains on the Arabian Peninsula may be possible in the future and should therefore be monitored.

To shed light on possible reasons for the restricted geographic circulation of different MERS-CoV strains, enhanced virological surveillance of MERS-CoV is urgently needed in dromedary populations of the affected regions. Putative underlying evolutionary and molecular mechanisms that influence the geographic distribution of differentially virulent MERS-CoV strains should be assessed through phenotypic characterizations of different MERS-CoV strains. The early detection and characterization of emerging MERS-CoV strains with new phenotypic features will be highly relevant for future vaccination strategies and the prediction of epidemics in humans.

## Electronic supplementary material

Supplementary material Kiambi & Corman
