# Supplementary material for: Detection of distinct MERS-Coronavirus strains in dromedary camels from Kenya, 2017
Source: Emerg Microbes Infect. 2018 Nov 28;7:195. doi: 10.1038/s41426-018-0193-z (PMC6258726; doi:10.1038/s41426-018-0193-z)
Supplement: Supplementary file 1 — Supplementary material Kiambi & Corman [file 41426_2018_193_MOESM1_ESM.docx]

**Supplementary Material**

**Supplementary Table.** Details of the MERS-CoV RNA-positive dromedary samples in Isiolo county collected April 20-21, 2017

| **Sample ID** | **Sex** | **Age** | **Sub location** | **Herd size** | **RNA concentration (RNA copies/ml)** | **Confirmation of MERS-CoV sequence** |
| --- | --- | --- | --- | --- | --- | --- |
| 011/LOM/C20/F<1 | Female | <1yr | Lombolio | 70 | 5.0E+07 | full genome |
| 011/DAB/C8/F<1 | Female | <1yr | Dabel | 128 | 3.7E+06 | full genome |
| 011/LOM/C19/F<1 | Female | <1yr | Lombolio | 70 | 7.6E+05 | SNP pattern of Lombolio LOM/C20# |
| 011/DAB/C11/M<1 | Male | <1yr | Dabel | 128 | 1.9E+05 | SNP pattern of DAB/C8# |
| 011/DAB/C7/F>3 | Female | >3yrs | Dabel | 128 | 1.2E+05 | SNP pattern of DAB/C8# |
| 011/DAB/C10/F>3 | Female | >3yrs | Dabel | 128 | 1,9E+04* | not done |
| 011/DAB/C13/F>3 | Female | >3yrs | Dabel | 128 | 1,9E+04* | not done |

#SNP: Detection of three single nucleotide polymorphisms in the spike and open reading frame 3 genomic region. Nucleotide genome positions according to MERS-CoV/EMC2012 (GenBank accession no. JX869059): 24,698 (Spike); 25,621 (ORF3), and 25,722 (ORF3).

*Presence of MERS-CoV could not be confirmed in a second real-time RT-PCR.

**Supplementary Figure**


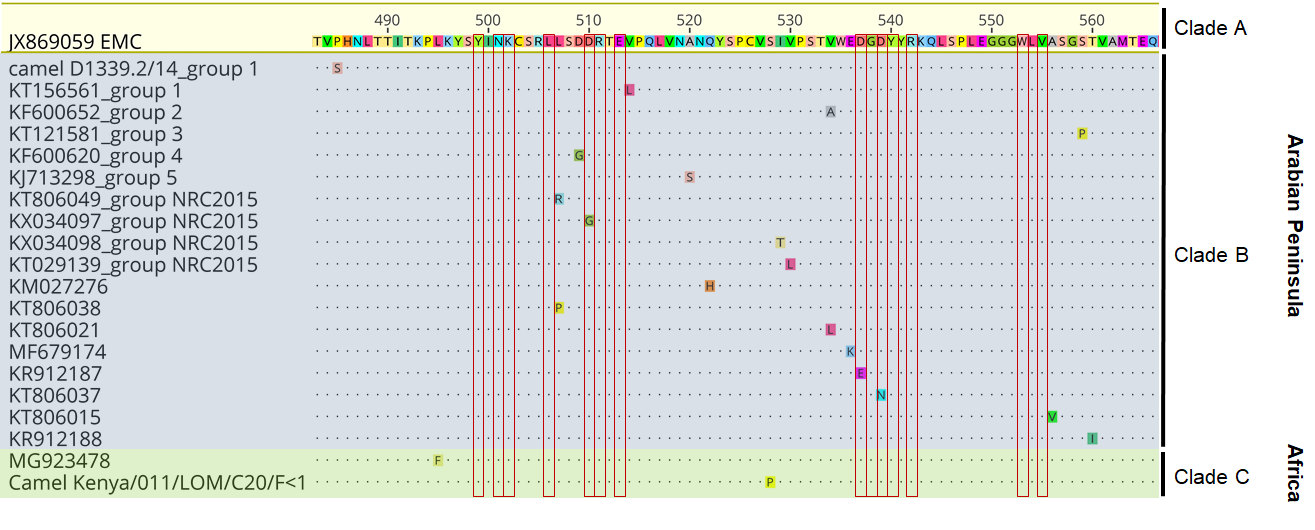


**Supplementary Figure. Amino acid alignment of the core part of the receptor-binding domain (RBD) of the MERS-Coronavirus spike protein.** In order to identify unique amino acid exchanges all published MERS-CoV spike sequences were initially aligned using the EMC strain as reference (not shown, Geneious version R9). In total, 91 MERS-CoV sequences had unique mutations within the core sequence of the RBD (amino acid positions 483-567). For clarity and to avoid redundancy, the figure includes either single sequences or examples of sequence clusters that have the same unique mutation. As both Kenyan MERS-CoV strains (Camel Kenya/011/LOM/C20/F<1 and Camel Kenya/011/DAB/C8/F<1) shared the S528P mutation, only one sequence was included (Camel Kenya/011/LOM/C20/F<1). The 14 essential amino acids responsible for direct interaction between the MERS-CoV spike RBD and the dipeptidyl peptidase 4 receptor are marked with red boxes.
